# The Orchid MADS-Box Genes Controlling Floral Morphogenesis

**DOI:** 10.1100/tsw.2006.321

**Published:** 2006-07-14

**Authors:** Wen-Chieh Tsai, Hong-Hwa Chen

**Affiliations:** ^1^ Department of Life Sciences, National Cheng Kung University, Tainan 701, Taiwan; ^2^ Institute of Biotechnology, National Cheng Kung University, Tainan 701, Taiwan

**Keywords:** orchids, MADS-box genes, ABCDE model, floral development, floral morphogenetic networks

## Abstract

Orchids are known for both their floral diversity and ecological strategies. The versatility and specialization in orchid floral morphology, structure, and physiological properties have fascinated botanists for centuries. In floral studies, MADS-box genes contributing to the now famous ABCDE model of floral organ identity control have dominated conceptual thinking. The sophisticated orchid floral organization offers an opportunity to discover new variant genes and different levels of complexity to the ABCDE model. Recently, several remarkable research studies done on orchid MADS-box genes have revealed the important roles on orchid floral development. Knowledge about MADS-box genes' encoding ABCDE functions in orchids will give insights into the highly evolved floral morphogenetic networks of orchids.

## INTRODUCTION

With more than 270,000 known species, angiosperms are by far the most diverse and widespread group of plants. The ancestry of the angiosperm is still uncertain. The fossil records show that the angiosperms appear at the early Cretaceous period, about 130 million years ago. By the end of the Cretaceous, 65 million years ago, the angiosperms had radiated and become the dominant plants on Earth, as they are today. The origin and diversification of angiosperms, what Charles Darwin characterized as "an abominable mystery", has been the subject of much speculation for the last 100 years[1,2,3]. The rapid explosion in diversity that followed their origin in the early Cretaceous may be linked to modularity within their new structure, the flower[4]. The flower is the defining reproductive adaptation of angiosperms, and is the predominant source of characters for angiosperm taxonomy and phylogeny reconstruction[5].

Over the past decades, the codification of rigorous methods of phylogenetic analysis, the emergence of molecular techniques, and a renewed interest in the developmental pathways followed during the growth of plant organs have improved understanding of angiosperm relationships[6,7,8,9]. The angiosperms consist of some small relic basal clades (basal angiosperms), magnollids, and two main clades: monocots and eudicots (Fig. 1). The basal angiosperms and magnollids share some primitive traits, such as a typical spiral rather than whorled arrangement of flower organs[10,11]. The monocots show extreme variation in floral form,

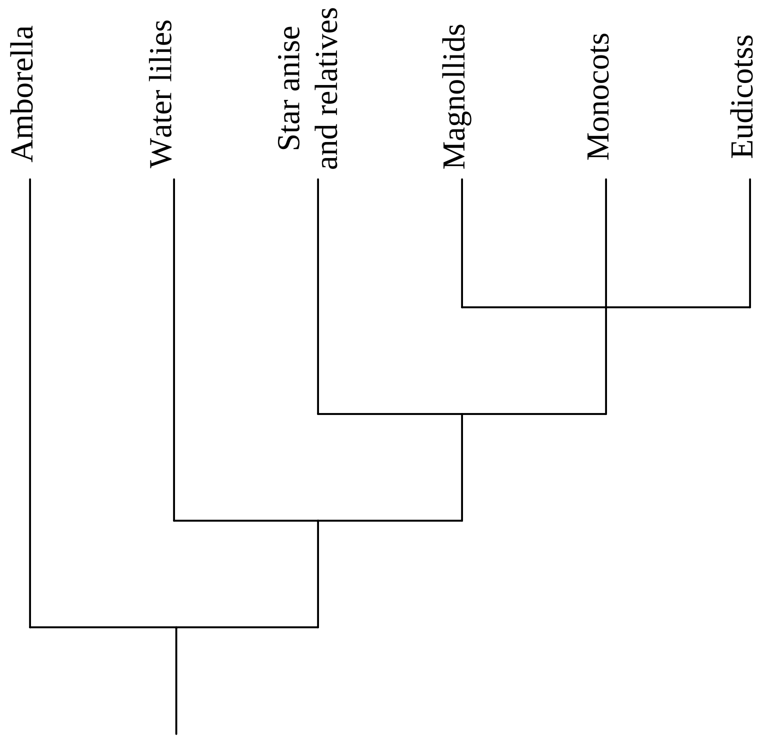

**FIGURE 1**. Phylogeny of angiosperms.

including bilaterally symmetric flowers with elaborately modified perianth parts. The organization of flower parts is a bit less variable in the core eudicots.

For plants, the MADS-box-containing transcriptional regulators have been the focus of floral organ specification, development, and evolutionary studies[12,13,14]. In the well-known "ABC model", the organ identity in each whorl is determined by a unique combination of three activities by organ identity genes, called A, B, and C[12]. In any one of the four flower whorls, expression of A alone specifies sepal formation. The combination of AB determines the development of petals and the combination of BC offers stamen formation. Expression of the C function alone determines the development of carpels. Functions of A and C are mutually repressive[15]. The ABC genes were cloned from a wide range of species and the model has been used to explain floral organ development in plants[15,16,17,18,19,20,21]. On the basis of studies on *Petunia*, the "ABC model" was later extended by including D-class genes that specify ovules[22]. An important recent discovery was that another set of MADS-box genes, *SEPALLATA1*, *2*, and *3*, function redundantly to specify petals, stamens, and carpels as well as floral determinacy[23]. Recently, *SEP4* has been defined. The *sep1 sep2 sep3 sep4* quadruple mutants develop vegetative leaves rather than sepals, petal, stamens, or carples[24]. SEPALLATA function, or so-called E function, has led to a revision of the "ABC model"' to become the "ABCDE model"[25,26]. The diversification of MADS-box genes during evolution has been proposed to be a major driving force for floral diversity in land plant architecture[15,27].

Containing more than 20,000 species, the Orchidaceae, classified in class Liliopsida, order Asparagales, is one of the largest angiosperm families. Associated with this enormous size is an extraordinary floral diversity. Orchids are extremely rich in species and speciation rates are presumed to be exceptionally high[28]. Although it has often been hypothesized that this spectacular diversification is linked to the intimate and sometimes bizarre interaction of many species with their pollinators[29], we are still facing the challenge of explaining how these mechanisms work and why they have evolved.

According to the classic view, the orchid flower is composed of five whorls of three segments each including two perianth whorls, two staminal whorls, and one carpel whorl (Fig. 2A)[30]. This also conforms to the general flower structure of many other monocotyledonous families. Orchidaceae represent an unusually coherent group among monocots, possessing several reliable floral morphological synapomorphies, including the presence of a gynostemium, or column, fused by the style and at least part of the androecium, a highly evolved petal called labellum, and resupination caused by 180$^o$ torsion of the pedicel[31]. Within the monocots, only well-known crop species, such as rice and maize, have been studied thoroughly, but the highly reduced flowers make them unsuitable for general floral development studies. All expected whorls in the flowers are present in orchids, and the highly sophisticated flower organization offers an opportunity to discover new variant genes and different levels of complexity within morphogenetic networks. Thus, the Orchidaceae can be used to test the validity of the "ABC model" in the monocots and to study how MADS-box genes are involved in defining the different highly specialized structures in orchid flowers.

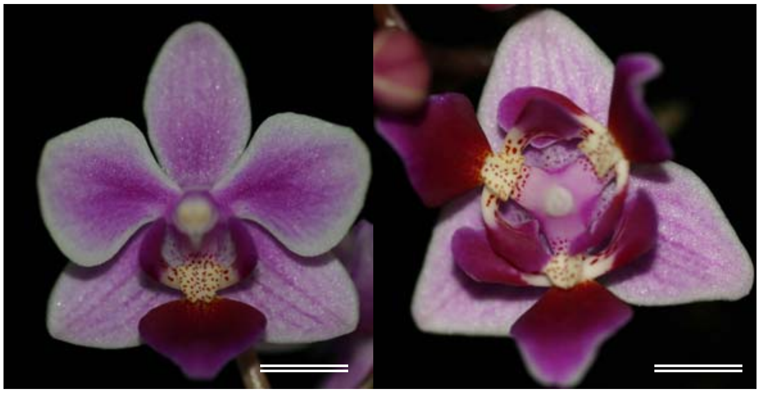

A B

**FIGURE 2.** Flowers from wild-type (A) and peloric mutant (B) of *Phalaenopsis equestris*. Bar = 1 cm.

## A-CLASS GENES IN ORCHIDS

To date, almost all cloned orchid MADS-box genes involved in floral development were from Epidendroideae, the largest orchid subfamily with many more genera and species than all of the other four subfamilies together (Table 1). So far, four A-class genes were identified from *Dendrobium*. One of them was isolated from *Dendrobium* grex Madame Thong-In, named as *DOMADS2*[32]. The other three genes were cloned from *D. thyrsiflorum*, named as *DthyrFL1*, *DthyrFL2*, and *DthyrFL3*, respectively[33]. The *APETALA1/FRUITFULL* (*AP1/FUL*) MADS-box gene lineage within the eudicots was recognized as two clades (the *euAP1* and *euFUL* clade), whereas the noncore eudicots and monocots only have sequences similar to *euFUL* genes[34]. Sequence analysis showed that these genes contain the C-terminal *FUL*-like motif LPPWML of monocot FUL-like proteins, but this motif is not present in the sequence of *DthyrFL3*[33]. Phylogenetic analysis showed that *DthyrFL1* and *DOMADS2* are orthologous genes (Fig. 3A). The existence of *DthyrFL2* and *DthyrFL3* represent a recent duplication event in *D. thyrsiflorum* (Fig. 3A[33]). *DOMADS2* is expressed in both of the shoot apical meristem and the emerging floral primordium throughout the process of floral transition and later in the column of mature flowers[32]. The expression pattern of *DOMADS2*, from shoot apical meristem and increasing in later stages of floral development, suggests that *DOMADS2* is one of the earliest regulatory genes during the transition of flowering. *DthyrFL* genes are expressed not only during inflorescence development, but also in developing ovules[33]. These A-class genes in orchids may be involved in floral meristem identity, and in

**TABLE 1**
**Floral MADS-Box Genes and Their Expression Patterns in Orchidaceae**

| Class | Gene | Species | Expression Pattern | | | | | | | | | Analysis |
|---|---|---|---|---|---|---|---|---|---|---|---|---|
| | | | Sepal | Petal | Labellum | Column | Pedicel/ Ovary | Root | Leaf | Shoot | Ovule | |
| A | *DOMADS2* | *Dendrobium* grex Madame Thong-In | –[a] | – | – | +[b] | – | – | – | + | ND[c] | I[d], II[e] |
| | *DthyrFL1* | *D. thyrsiflorum* | ND | ND | ND | ND | ND | – | – | ND | + | III[f] |
| | *DthyrFL2* | *D. thyrsiflorum* | ND | ND | ND | ND | ND | – | – | ND | + | III |
| | *DthyrFL3* | *D. thyrsiflorum* | ND | ND | ND | ND | ND | – | – | ND | + | III |
| B (paleo*AP3*) | *OMADS3* | *Oncidium* Gower Ramsey | + | + | + | + | ND | ND | + | ND | ND | IV[g] |
| | *PeMADS2* | *Phalaenopsis equestris* | + | + | – | – | – | – | – | – | ND | I |
| | *PeMADS3* | *P. equestris* | – | + | + | – | – | – | – | – | ND | I |
| | *PeMADS4* | *P. equestris* | – | – | + | + | – | – | – | – | ND | I |
| | *PeMADS5* | *P. equestris* | + | + | + | – | – | – | – | – | ND | I |
| | *DcOAP3A* | *D. crumenatum* | + | + | + | + | + | ND | + | ND | ND | II, IV |
| | *DcOAP3B* | *D. crumenatum* | – | + | + | + | + | ND | – | ND | ND | IV |
| B (PI) | *PeMADS6* | *P. equestris* | + | + | + | + | + | – | – | – | ND | I, II |
| | *DcOPI* | *D. crumenatum* | + | + | + | + | + | ND | – | ND | ND | II, IV |
| | *ORCPI* | *Orchis italica* | ND | ND | ND | ND | ND | ND | ND | ND | ND | |
| C | *PeMADS1* | *P. equestris* | – | – | – | + | + | – | – | – | ND | I, IV |
| | *PhalAG1* | *Phalaenopsis* Hatsuyuki | – | – | – | + | + | ND | ND | ND | + | II, IV |
| | *DthyrAG1* | *D. thyrsiflorum* | – | – | – | + | ND | ND | ND | ND | + | II, III |
| | *DcOAG1* | *D. crumenatum* | + | + | + | + | + | ND | – | ND | ND | II, IV |
| D | *PhalAG2* | *Phalaenopsis* Hatsuyuki | – | – | – | + | + | ND | ND | ND | + | II, IV |
| | *DthyrAG2* | *D. thyrsiflorum* | – | – | – | + | ND | ND | ND | ND | + | II, III |
| | *DcOAG2* | *D. crumenatum* | – | – | – | + | + | ND | – | ND | ND | II, IV |
| E | *OM1* | *Aranda* Deborah | + | + | + | - | ND | ND | ND | ND | ND | I |
| | *DOMADS1* | *Dendrobium* grex Madame Thong-In | + | + | + | + | + | – | – | – | ND | I, II |
| | *DOMADS3* | *Dendrobium* grex Madame Thong-In | – | – | – | – | + | – | – | – | – | I, II |
| | *DcOSEP1* | *D. crumenatum* | + | + | + | + | + | ND | - | ND | ND | IV |

[a] Transcripts of gene were not detected.
[b] Transcripts of gene were detected.
[c] Detection of transcripts has not been performed.
[d] Transcripts were detected by Northern blotting.
[e] Transcripts were detected by *in situ* hybridization.
[f] Transcripts were detected by real-time RT-PCR.
[g] Transcripts were detected by RT-PCR.

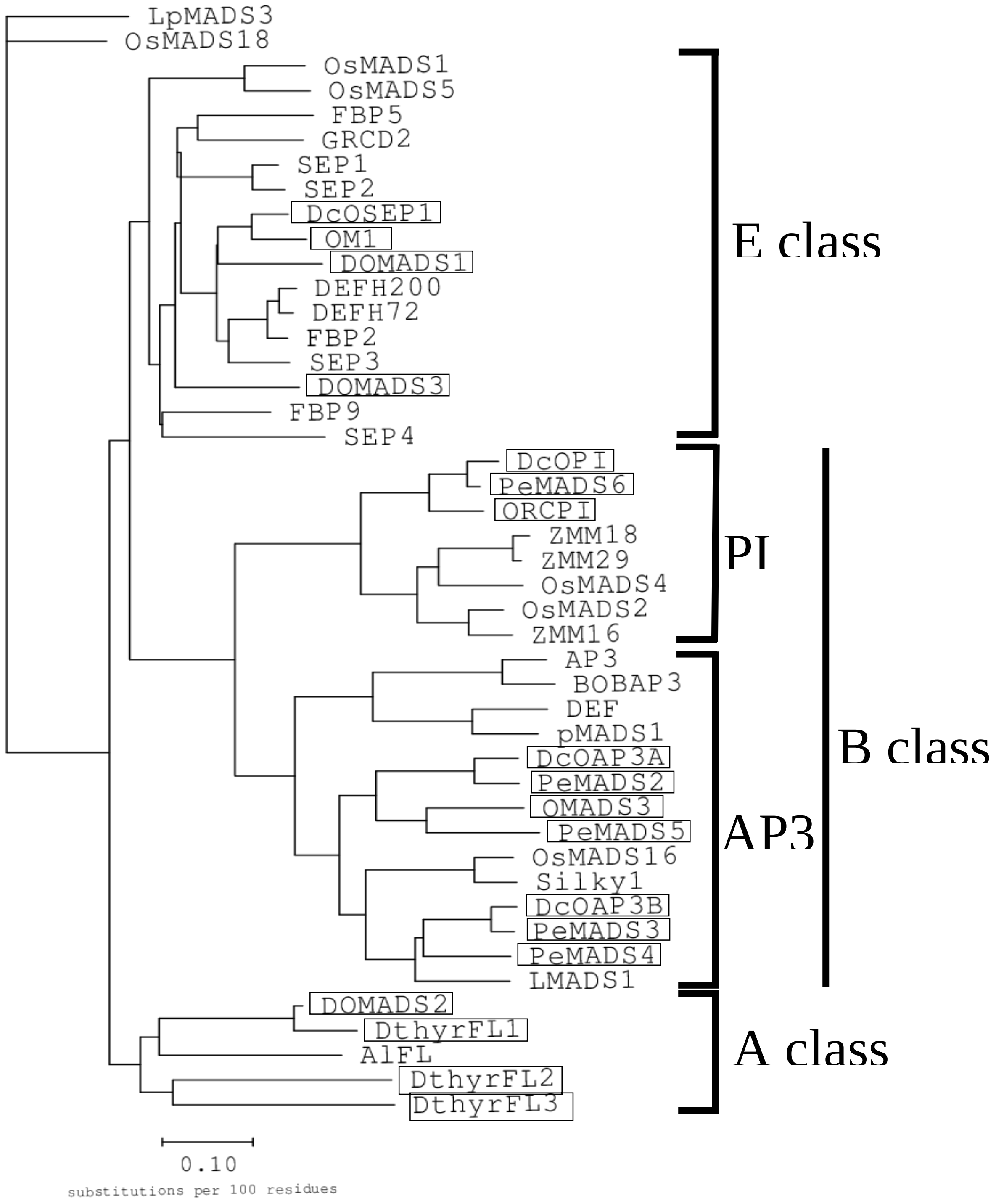

**A**

**FIGURE 3**. Phylogenetic relationship of MADS-box genes of ABCDE class. (A) Phylogenetic analysis of A-, B-, and E-class genes. (B) Phylogenetic analysis of C- and D-class genes. Orchid MADS-box genes are highlighted by open boxes.

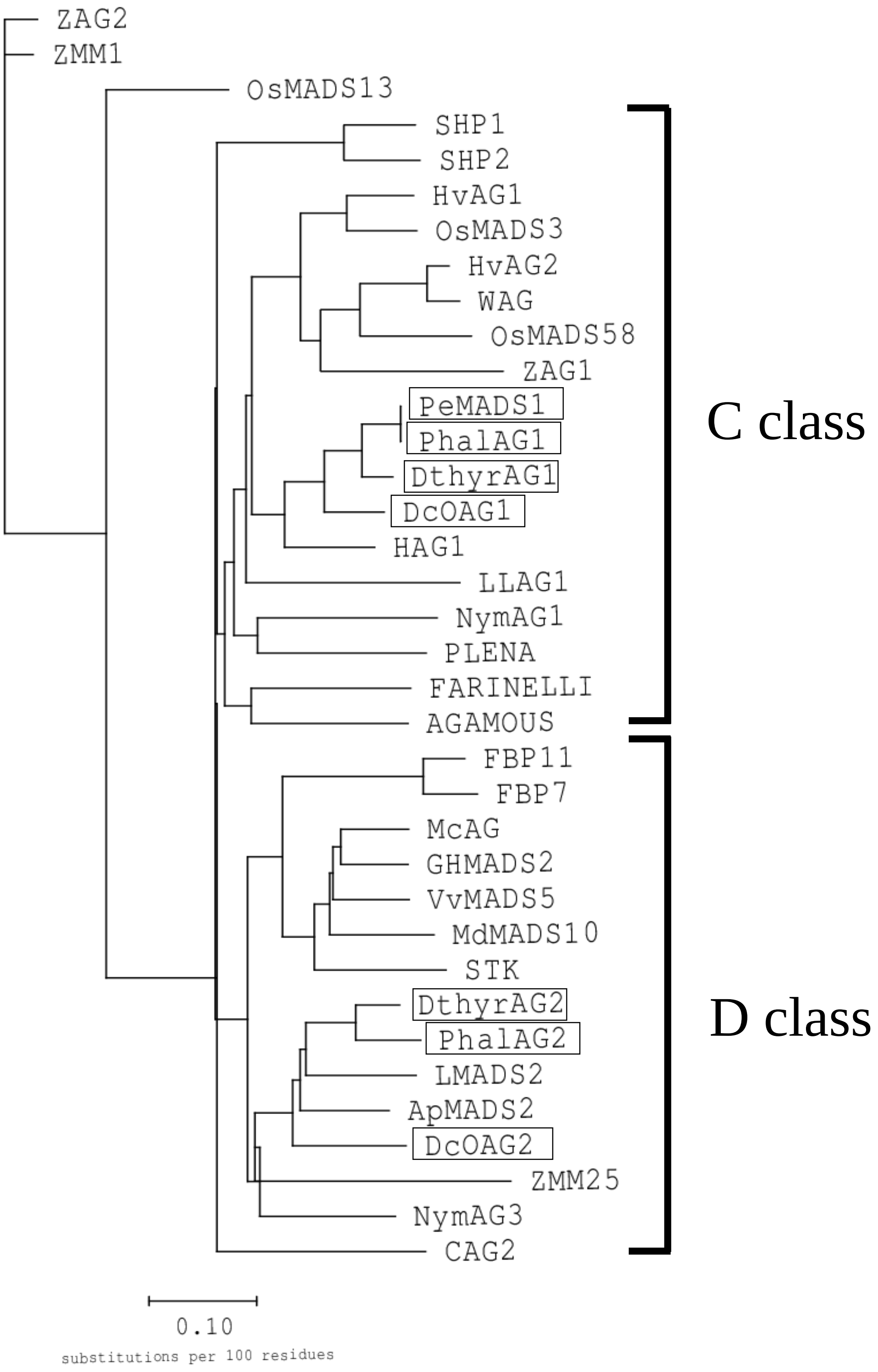

ZAG2
ZMM1
OsMADS13
SHP1
SHP2
HvAG1
OsMADS3
HvAG2
WAG
OsMADS58
ZAG1
PeMADS1
PhalAG1
DthyrAG1
DcOAG1
HAG1
LLAG1
NymAG1
PLENA
FARINELLI
AGAMOUS
FBP11
FBP7
McAG
GHMADS2
VvMADS5
MdMADS10
STK
DthyrAG2
PhalAG2
LMADS2
ApMADS2
DcOAG2
ZMM25
NymAG3
CAG2
C class
D class
0.10
substitutions per 100 residues

**FIGURE 3B**

column and ovule development. Unlike its homolog *AP1* in *Arabidopsis*, the *DOMADS2* may not correlate with the development of the first two whorls. However, it is not clear whether the *DthyrFL* genes associate with perianth formation. In addition, a MADS-box gene *OMADS1* in the *Oncidium* orchid, also involved in floral initiation and formation, belongs to the *AGL6* subfamily rather than the A-class gene[35]. Transgenic *Arabidopsis* and tobacco overexpressing *OMADS1* showed significantly reducing plant size, extremely early flowering, and losing inflorescence indeterminacy[35].

## B-CLASS GENES IN ORCHIDS

Both the developmental and the biochemical aspects of B-class genes required to specify the identity of petals in whorl 2 and stamens in whorl 3 appear to be conserved in many core eudicots[15,16]. The B-class genes in monocots rice and maize are similar in function to the core eudicot B-class genes[19,20,36,37]. The orchid flowers have a petaloid perianth arrangement that could be explained by a "modified ABC model" in that the expression of the B-class genes has expanded to whorl 1[38]. In addition, the orchid flowers display an elaborated labellum that is a highly modified petal. As the function of A-class genes is poorly defined in angiosperms, studies of petal development and evolution have generally focused on B-class genes[39]. The extraordinary floral diversity in orchids may be correlated with the evolution of B-class genes.

Molecular functions of B-class genes were studied in more detail than any other floral homeotic genes in orchids. So far, various numbers of *APETALA3* (*AP3*)-like and *PISTILLATA* (*PI*)-like genes have been isolated from several orchids. These include one *AP3*-like *OMADS3* isolated from *Oncidium* Gower Ramsey, four *AP3*- and one *PI*-like genes identified from *Phalaenopsis equestris*, and two *AP3*- and one *PI*-like genes cloned from *Dendrobium crumenatum*[40,41,42,43]. All these *AP3*-like genes in orchids are the members of the paleo*AP3* lineage (Fig. 3A). The paleo*AP3* genes identified from orchids were subdivided into two subclades. One subclade contains *OMADS3*, *PeMADS5*, *DcOAP3A*, and *PeMADS2*, while *OcOAP3B*, *PeMADS3*, and *PeMADS4* make up the other subclade (Fig. 3A). This result suggests that the ancestor of Orchidaceae might have had two paleo*AP3*-like genes, and further gene duplication has at least taken place in the *AP3* clade in the monocots. Interestingly, both *OMADS3* and *PeMADS5* do not present obvious paleoAP3 motif, suggesting that they are orthologous genes. Although they share similar expression patterns in orchid floral organs, *PeMADS5* is not expressed in vegetative tissues, but the expression of *OMADS3* can be detected in leaves[40,41]. Phylogenetic analysis also showed that *DcOAP3A* and *PeMADS2* are orthologous genes (Fig. 3A). However, they possess different expression patterns. Similar to *OMADS3*, the *DcOAP3A* is ubiquitously expressed in all floral organs and in leaves, while *PeMADS2* is predominantly expressed in sepals and petals[40,41,43]. Recently, we discovered that there were at least three paleo*AP3* genes displaying distinct expression patterns in *Oncidium* floral organs (our unpublished data). In addition, we also noticed that the expression profile of *OMADS3* examined by Hsu and Yang[40] was indeed composed of the expression patterns from two *paleo*AP3 genes of *Oncidium* (our unpublished data). Distinctly, a specialized paleo*AP3* gene, *PeMADS4* discovered in *P. equestris*, is specifically expressed in labellum and column, suggesting an association of its function with the development of orchid labellum and column. Gene duplication is important for generating new genes during evolution[44], and thus may lead to the generation of new organs. In orchids, duplication of paleo*AP3*-like genes, followed by diversification and specialization probably is associated concomitantly with the arising of new floral organ, labellum, in orchids.

Overexpression of *paleo*AP3 genes from *Oncidium*, *Dendrobium*, and *Phalaenopsis* under the control of the cauliflower mosaic virus 35S promoter was examined in *Arabidopsis*[40,43,our unpublished data]. Consistently, all these results showed that the flower morphology of the transgenic *Arabidopsis* plants overexpressing the orchid *paleo*AP3 genes are indistinguishable from that of the wild-type plants. Dominant negative mutation strategy was further conducted to investigate the functions of *OMADS3* and *DcOAP3A*[43]. By doing this, the *OMADS3* has been shown to have a function similar to the A functional gene in regulating flower formation as well as floral initiation, whereas the *DcOAP3A* has a putative B

function[40,43]. However, these results could not reflect the real roles they may play during orchid floral development.

The peloric flowers that are actinomorphic mutants with lip-like petals are widely found in natural populations of species from Veronicaceae, Gesneriaceae, Labiatae, and Orchidaceae[45]. With the presence of a high frequency of orchid peloric mutants derived from micropropagation (Fig. 2B), we were able to infer the individual roles played by the diversified *paleo*AP3 genes in orchids by comparing the expression patterns of the four *paleoAP3* (*PeMADS2*, *PeMADS3*, *PeMADS4*, and *PeMADS5*) genes in wild-type *Phalaenopsis* floral organs and in peloric mutants[41]. First, we discovered that both *PeMADS2* and *PeMADS5* were expressed in the sepal of wild-type plants, but only *PeMADS2* transcript was detected in the sepal of the peloric mutant whose morphology is not affected. This result suggests that *PeMADS5* is dispensable, while *PeMADS2* is crucial for the sepal development. Second, the expressions of all four *PeMADS* genes, except *PeMADS4*, were detected in the wild-type petals. However, the expression of *PeMADS5* was not noted in the lip-like petals of the peloric mutant. This result suggests that *PeMADS5* correlates with the petal development. Third, the expression of *PeMADS4* is concentrated in lips and columns in the wild-type plant**,** and is extended to the lip-like petals in the peloric mutant. The fact that the *PeMADS4* transcript was detected in the lip-like petal of the peloric mutant suggests that *PeMADS4* is required for labellum identity. Fourth, the *PeMADS3* shows similar expression patterns in the wild-type plant and the peloric mutant, suggesting its important function in inner perianth whorl morphogenesis.

So far, there is only one *PI*-like gene found in *D. crumenatum* and *P. equestris*, *DcOPI* and *PeMADS6*, respectively[42,43]. The Southern blot hybridization results supported that the *Phalaenopsis* orchid genome contains only one copy of the *PI*-like gene[42]. Both genes are expressed in all floral organs, except that *PeMADS6* is not detected in the pollinia of *P. equestris*[42,43]. In addition, the *PeMADS6* is expressed in the undeveloped ovary[42]. Tsai et al.[42] suggested that the expression of *PeMADS6* in ovary has an inhibitory effect on the development of the ovary, and auxin acts as the candidate signal to regulate the repression of *PeMADS6* expression in the ovary. Furthermore, the *PeMADS6* is not differentially expressed between wild-type and peloric floral organs, which suggests that *PeMADS6* is not responsible for the altered phenotype of the peloric mutant. Overexpression of *DcOPI* or *PeMADS6* in *Arabidopsis* demonstrated that both of them share the angiosperm *PI* function[42,43]. Further evidence came from the complementation of *pi-1* phenotype in *Arabidopsis* by overexpressing *DcOPI* and showed that *DcOPI* is able to substitute *PI* in *Arabidopsis*[42], while *PeMADS6* could not complement *pi-4* mutant (our unpublished data).

In conclusion, expression patterns of B-class genes in orchid floral organs nicely fit the “modified ABC model” in that the expression of the B-class genes has expanded to whorl 1 in plants possessing nearly identical morphology of sepals and petals[38]. paleo*AP3* genes are highly duplicated in Epidendroideae genome. Diversification and fixation of both these gene sequences and expression profiles might cause the subfunctionalization and even neofunctionalization. Driving force of specialized labellum and diversified orchid flowers may be linked to the fast evolution rate of paleo*AP3* genes. Study of the B-class genes from other orchid subfamilies, such as Apostasioideae, Cypripedioideae, Spiranthoideae, Orchidoideae, and even more members of Epidendroideae[46], will provide profound knowledge to resolve.

## C- AND D-CLASS GENES IN ORCHIDS

A gynostemium or column, comprising stamen filaments adnate to a syncarpous style, is normally regarded as a structure peculiar to the orchids[46]. The development of a column, which involves whorl 3 and whorl 4, would be one of the most interesting subjects to elucidate the evolution of C-class genes. In most orchid flowers, ovary and ovule development is precisely and completely triggered by pollination, and thus orchids offer a unique opportunity to study D-class genes involving ovule development. More recently, one C-class gene and one D-class gene were isolated independently from three orchid species,

*Phalaenopsis* Hatsuyuki (*PhalAG1*, *PhalAG2*[47]), *D. thyrsiflorum* (*DthyrAG1*, *DthyrAG2*[48]), and *D. crumenatum* (*DcOAG1*, *DcOAG2*[43]). *PhalAG1*, *DthyrAG1*, *DcOAG1* were classified in C-lineage of AG-like genes, and *PhalAG2*, *DthyrAG2, DcOAG2* were classified in D-lineage of AG-like genes (Fig. 3B). The *PhalAG1*, *PhalAG2*, *DthyrAG1*, and *DthyrAG2* share very similar spatial expression patterns in column, ovary, and developing ovules despite the fact that these four genes belong to different lineages[47,48]. One possible explanation is that C- and D-class genes in orchids would act redundantly with each other in floral and ovule development. Although *PhalAG1* is expressed in all floral organs at their initiation, its expression quickly decreases and then can only be detected in column and ovary when flowers mature, and this is also true for *DthyrAG1*[47,48]. We also identified a C-class gene, *PeMADS1*, from *P. equestris*, and expression patterns investigated were consistent with *PhalAG1* and *DthyrAG1* (our unpublished data). However, *DcOAG1* is expressed in all mature floral organs and *DcOAG2* is expressed in anther cap and column of *D. crumenatum*[43]. The unusual expression patterns of *DcOAG1* in monocots evoke that the regulatory mechanism of *DcOAG1* is independently evolved in *D. crumenatum* as some basal angiosperms, such as *Illicium* and *Persea*[49], but the function of *DcOAG1* and *Arabidopsis AG* is conserved as supported by the phenotypic similarity between transgenic *Arabidopsis* expressing either *35S::DcOAG1* or *35S::AG*[43]. Molecular mechanism of morphogenesis of orchid gynostemium is still enigmatic. Mutation of C-class genes in orchids could possibly provide the opportunity to shed light on the mystery.

## E-CLASS GENES IN ORCHID

E-class genes are required for floral organ identity in all four floral organs as well as floral determinacy[24,50]. They have been shown to form ternary complexes with A-class proteins and with B-class proteins in yeast three-hybrid system and can mediate the interactions between B- and C-class proteins in higher-order complexes[51]. The first E-class gene, *OM1,* was isolated from the supposed bigeneric hybrid *Aranda* Deborah[52]. The other three E-class genes (*DOMADS1*, *DOMADS3*, *DcOSEP1*) were identified from *Dendrobium*; two of them were cloned from *Dendrobium* grex Madame Thong-In, the other one was isolated from *D. crumenatum*[32,43]. Phylogenetic analysis showed that *OM1* was clustered with *DOMADS1* and *DcOSEP1*, and *DOMADS3* separated itself from the other three orchid E-class genes at a distance (Fig. 3A). *DOMADS1* RNA is uniformly expressed in both of the inflorescence meristem and floral primordium and later existed in all of the floral organs[32]. The expression pattern of *DOMADS1* in mature flowers coincides with its counterpart *DcOSEP1* in *D. crumenatum*, as with their orthologs in *Arabidopsis*[23,32,43]. However, *OM1* is expressed in mature flowers and not in young developing inflorescence or young floral buds. In the mature flowers, it is only expressed in petals and weakly in sepals, but not in the column[52]. Spatiotemporal expression differences imply that functional diversification among these genes closely relates in phylogeny. The onset of *DOMADS3* transcription is in early shoot apical meristem at the stage before the differentiation of the first flower primordium, and later can only be detected in the pedicels[32]. The *DOMADS3* may function as a regulatory factor not only in early floral transition, but also in the development of the pedicel. The expression of E-class genes overlapping with ABC genes in orchids suggests that the higher-order MADS complexes are involved in orchid floral development. Recently, one line of evidence that MADS proteins form higher-order complexes comes from the formation of DcOAP3A-DcOPI-DcOSEP1 and DcOAP3B-DcOPI-DcOSEP1 was detected by using yeast three-hybrid experiments[43].

## PERSPECTIVE

Owing to the large genome size, long life cycle, and inefficient transformation system of orchids, a few studies for orchid biology exist. Recently, the genetic architecture of orchid-sophisticated floral organization has begun to be investigated. Given that the orchids represent the most successful and diverse plant families worldwide, the development of genomic resources is an imperative[53]. Thank to the advanced progress of genomics and bioinformatics, plentiful gene information and integrated bioinformatic tools are ready to be used for studying orchid biology[54,55,56]. The effort of many scientists will promise to lead to a better understanding of the molecular and genetic mechanisms of orchid floral control in the years to come.

## ACKNOWLEDGMENT

We thank Wen-Huei Chen (Department of Life Sciences, National Science Council, National University of Kaohsiung, Kaohsiung, Taiwan) for leading us into the field of orchids. We thank Chang-Sheng Kuoh (Department of Life Sciences, National Cheng Kung University, Tainan, Taiwan) for sharing his knowledge of orchid floral ontogeny. We thank Tung-Hua David Ho (Institute of Plant and Microbial Biology, Academia Sinica, Taipei, Taiwan) and Michel Delseny (Laboratory of Plant Genome and Development, University of Perpignan, France) for helpful discussions. Grant funding from both the National Science Council and Council of Agriculture in Taiwan support the current research in our labs.

## BIOSKETCH

**Hong-Hwa Chen** is a professor in the Department of Life Sciences at National Cheng Kung University, and **Wen-Chieh Tsai** is currently a postdoctoral researcher in Dr. Chen’s lab. They have worked on *Phalaenopsis* orchids for 10 years. They apply genomics approaches to study the nonmodel plants with large genome size by establishing EST databases, and focus on the B-class genes for orchid floral development. In addition, they have collaborated with colleagues on Chemistry and Information Management to reveal the orchid fragrance metabolic pathways.
